# Why does the provision of home mechanical ventilation vary so widely?

**DOI:** 10.1177/1479972309357497

**Published:** 2010-05

**Authors:** Knut Dybwik, Terje Tollåli, Erik Waage Nielsen, Berit Støre Brinchmann

**Affiliations:** 1 Department of Anesthesiology, Nordland Hospital, Bodø, Norway; 2 Medical Department, Nordland Hospital, Bodø, Norway; 3 Institute of Clinical Medicine, University of Tromsø, Norway; 4 Faculty of Professional Studies, Bodø University College, Norway

**Keywords:** enthusiasm, focus groups, grounded theory, home mechanical ventilation, provision

## Abstract

There is wide variation in the provision of home mechanical ventilation (HMV) throughout Europe, but the provision of home mechanical ventilation can also vary within countries. In 2008, the overall prevalence of HMV in Norway was 19.9/100,000, and there were huge regional differences in treatment prevalence. The aim of this study is to find explanations for these differences. We gathered multidisciplinary respondents involved in HMV treatment from five hospitals in five different counties to six focus group conversations to explore respondents' views of their experiences systematically. We based the analysis on grounded theory. We found that uneven distribution of “enthusiasm” between hospitals seems to be an important factor in the geographical distribution of HMV. Furthermore, we found that the three subcategories, “high competence,” “spreading competence,” and “multidisciplinary collaboration,” are developed and used systematically in counties with “enthusiasm.” This culture is the main category, which might explain the differences, and is described as “wise enthusiasm.” The last subcategory is “individual attitudes” about HMV among decision-making physicians. The most important factor is most likely the uneven distribution of highly skilled enthusiasm between hospitals. Individual attitudes about HMV among the decision makers may also explain why the provision of HMV varies so widely. Data describing regional differences in the prevalence of HMV within countries is lacking. Further research is needed to identify these differences to ensure equality of provision of HMV.

## Introduction

The Universal Declaration of Human Rights states that all humans are legally obligated to have fair and equal access to health care. The Eurovent survey of home mechanical ventilation (HMV) in Europe showed a substantial variation between countries in the likelihood of patients with chronic respiratory failure receiving HMV,^1^ but the provision of home mechanical ventilation can also vary within each country. In 2008, the national treatment prevalence for home mechanical ventilation in Norway was approximately 19.9/100,000.^2^ There are considerable regional differences in treatment prevalence of HMV,^2,3^ despite the fact that the prevalence of conditions leading to chronic ventilatory insufficiency is unlikely to vary significantly. To date, there has been no research on the causes of these differences. Other than Scandinavia, there is lack of data describing regional differences in prevalence of HMV within countries. Studies from Sweden have also revealed large regional differences in treatment prevalence of HMV.^4,5^ In these studies, no systematic research into the causes of these differences was conducted, however, the authors have suggested some likely explanations. A statistically significant connection between the relative number of pulmonologists and the number of home-ventilated patients was presented as a possible explanation.^5^ Laub et al. maintained that the most likely explanation could be attributed to the individual physician, due to the variations in levels of ambitions and extent of problem discovery, and to the fact that high prevalence counties had dedicated clinicians.^4^ Bach discovered substantial differences between American clinics in regard to prescription of home mechanical ventilators for patients with muscular dystrophy.^6^ The study concluded that clinic managers who rejected starting with HMV, to a larger extent, regarded the quality of life for home mechanical ventilation patients to be lower compared to those who recommended treatment.

In order to take measures to even out regional differences within each country, it is important to have knowledge about what causes these differences. The aim of this study is to find explanations for the vast differences in treatment prevalence of HMV between the Norwegian counties. By using qualitative methods, we investigated the experiences of health personnel involved in HMV, which may explain these differences.

## Method

Pubmed, Google Advanced Search and Google Scholar were used to identify regional differences in prevalence of HMV within countries. We gathered multidisciplinary respondents from five Norwegian hospitals in five different counties for focus groups. Focus groups were used for this qualitative study because they have proven to be particularly useful for gaining thorough descriptions of knowledge, experience, priorities, and attitudes.^7,8^ The spontaneity of group-based conversations may provide insight into topics that are difficult to gain by using other methods.^9^ The focus groups were conducted between August 2008 and February 2009, and lasted between 70 and 90 minutes. We did not decide on the number of focus groups initially, but stopped interviewing when we discovered no new data or variables emerged from the conversations. This “theoretical saturation” is in accordance with the grounded theory method used for data analysis.^10^ Altogether we carried out six focus groups because two interviews were conducted in one specific county to gather all relevant respondents. All respondents were involved in the establishment and follow-up of HMV patients and were recruited through local contact persons at each hospital. The professions of the 34 respondents are described in Table 1. We conducted the interviews at hospitals in counties with treatment prevalence varying from 24 to 58 (Table 1). One of the hospitals is a university (teaching) hospital (focus group 5), and the four remaining are medium-sized hospitals.

A moderator led the conversations, which were steered according to a discussion guide containing a few open questions. To gain in-depth insight into the descriptions that were most important to the respondents and that often reappeared in the data material, we adjusted the discussion guide for each focus group based on on-going analyses and comparisons of the gathered data. Continuous notations where recorded by a secretary. The focus groups' conversations were recorded and transcribed verbatim. Thoughts and ideas (memos) were noted during the conversations. Printouts and written memos were analyzed line by line to find words or phrases used by the respondents to describe the causes of HMV differences between the counties, which is also known as open coding. At a later stage, we sorted the codes into larger categories and subcategories. We found a main category, which was most important to the respondents in this study and which often reappeared in the data material. Furthermore, we found four subcategories that were associated with the main category.

The study was approved by the Norwegian Social Science Data Services (09/22/08, 19729/2/JE) and Regional Committee for Medical Research Ethics (REKNORD 98/2008). Participating hospitals were informed through information letters, and all respondents gave their written consent to participate. No respondents received monetary compensation for participating in the focus groups. All gathered material has been treated anonymously.

## Results

We found that uneven distribution of “enthusiasm” between hospitals seems to be an important factor in the geographical distribution of HMV. Furthermore, we found that the three subcategories, “high competence,” “spreading competence,” and “multidisciplinary collaboration” are developed and used systematically in counties with “enthusiasm.” This culture is the main category that might explain the differences and is described as “wise enthusiasm.” The last subcategory is “individual attitudes” about HMV among decision-making physicians.

### Wise enthusiasm (main category)

Home mechanical ventilation is established by a few experienced and highly skilled enthusiasts, especially in counties with high treatment prevalence. Enthusiasts consisted primarily of physicians, specifically pulmonologists and anesthesiologists, but were also physician-supported nurses. Enthusiasts were defined as health care providers that possessed a special interest in HMV and a willingness to invest in the patients with documented effects of HMV. Enthusiasm was regarded as necessary and commendable, but too much enthusiasm could lead to the admittance of too many patients at some hospitals (Table 2).

### High competence

The need for high competence in HMV was significant for patient recruitment and patient recruitment was dependent on the availability of other highly competent health care providers at each hospital. HMV is a relatively rare and highly specialized treatment, and fairly little known in the health care system, both within and outside the hospital. The enthusiast must therefore be accompanied by a multidisciplinary team that is highly competent as well (Table 3).

**Table 1.** Characteristics of participating hospitals and respondents

Focus group	Treatment prevalence in counties we interviewed	Profession
1	57	Nurses, pulmonologist
2	31	Nurses, pulmonologist, anesthesiologist, neurologist, medical device technician
3	58	Nurse, pulmonologist, anesthesiologist, neurologist, pediatrician
4	38	Nurse, physiotherapists, pulmonologist, pediatrician, medical device technician
5	24	Nurses, pulmonologist, anesthesiologist, ENT doctor, secretary
6	57^a^	Pulmonologist, anesthesiologist, pediatricians, neurologist

a Same hospital as focus group 1.

**Table 2.** Quotation examples ‘wise enthusiasm’

‘It is obvious, if there is an enthusiastic environment that gives high priority to HMV, and organizes it, then this will be the main difference, in my opinion.’ (Physician)	‘You've got to have a passion for it. If you are to do a good job as a physician, regardless of area, passion and willingness to go the extra mile is essential. You have to be interested in that subject area.” (Physician)	‘I believe it's got to do with the fact that the treatment is fairly special, so you need eager persons who are pioneers.’ (Physiotherapist)	‘If we end up with an attitude of “more is better,” I think we're making a mistake.’ (Physician)

**Table 3.** Quotation examples ‘high competence’

‘Qualified and actively working HMV personnel are an important factor. Where there is a lack of knowledge, you will not find these patients.’ (Physician)	‘It's got to do with the fact that a certain competence level is needed in order to make it work. That's what we have, and therefore we are probably at a high level.’ (Physician)	‘If you are to start with home mechanical ventilation without having done so before ... it is almost impossible. ... so if you have a pulmonologist with no experience in this area, I can't believe it will turn out OK.’ (Physician)	‘I believe it's rather important that the responsibility lies with certain centers because there are relatively few patients. Experience is a must. You will not become competent if you do not practice the work.’ (Physician)

### Spreading competence

In addition to health care services outside the hospital to discover and recruit new patients, respondents also emphasized the importance of spreading competence within the different departments of the hospitals. Some of the hospitals offered courses for health care personnel from primary care in the community and other hospitals (Table 4).

**Table 4.** Quotation examples ‘spreading competence’

‘Professionals have visited our hospital and seen what we do, the way we think and what we have to offer. This made it possible for them to return to their local hospital and put into practice what they have learned from us.’ (Nurse)	‘Over the last few years we've had very structured and massive arrangement in regard to training, courses and contact with the county council, both before and after the patient have been transferred to the community.’ (Physician)	‘Counties with few patients have had the greatest benefit of the Norwegian Medical Center for Home Mechanical Ventilation.’ (Physician)

### Multidisciplinary collaboration

Respondents reported that a close and systematic multidisciplinary collaboration was significant for the quality and quantity of the HMV. This collaboration is important to detect potential patients at an early stage and to support the enthusiasts. Multidisciplinary collaboration was also particularly significant in difficult ethical decisions (Table 5).

**Table 5.** Quotation examples ‘multidisciplinary collaboration’

‘But we have done this systematically. We do not experience any problems with our multidisciplinary collaboration among the different disciplines here at our hospital, due to the fact that we all know each other.’ (Nurse)	‘I think the collaboration we've had has worked well and might also have been a contributing factor to why more patients have been offered the treatment. This is why we've managed to collaborate with the neurologist and why we've gotten involved with the patients at an earlier stage.’ (Nurse)	‘It also depends on a rather well-functioning collaboration between all parties, such as neurologists, anesthesiologists and pulmonologists. I guess we feel that we don't collaborate very well with the neurologist. I think they have withdrawn slightly from it.’ (Physician)

### Individual attitudes

Respondents emphasized that individual attitudes towards HMV had great significance in whether patients were offered treatment or not. Particularly crucial were the individual attitudes of decision-making neurologists. Offering life-prolonging and resource-demanding home mechanical ventilation was regarded controversial by some (Table 6).

**Table 6.** Quotation examples ‘individual attitudes'

‘Neuro has been very nihilistic to many of their diagnosis groups. Pediatrics also. ... If you have chosen not to provide such a comprehensive and controversial treatment, and accepted that it won't work, then it won't work.’ (Physician)	‘If you live in a very nihilistic environment over a long period of time, it is obvious that you become nihilistic, too, and then the indication for giving such a treatment may vary in the different hospitals.’ (Physician)	‘In their opinion, it is not ethically correct to prolong life in patients with diseases like ALS, who end up tied to their beds without being able to move with a ventilator. The physician doesn't think this is a worthy life, and therefore no measures are taken.’ (Physician)

When the respondents in the focus groups were asked to explain the differences between the counties, we discovered that they were talking about experiences from their own hospital. The respondents also had thorough knowledge about subject persons at other hospitals, how many patients these hospitals had, which diagnostic groups the hospitals prioritized and how the hospitals organized the treatment. An explanation to this may be that many of the respondents were collaborators in a national network employed by the Norwegian Medical Center for Home Mechanical Ventilation.

## Discussion

We found that an uneven distribution of wise enthusiasm between hospitals seems to be an important factor in the geographical distribution of HMV. We also found that individual attitudes about HMV among decision-making physicians may lead to an uneven distribution of such a highly specialized treatment.

The respondents in our research regarded HMV as a relatively new and highly specialized treatment for relatively few patients. Establishing and providing such a specialized treatment required special competence. In high prevalence counties HMV is, to a great extent, established by a small number of experienced enthusiasts with high competence. In their study of the huge differences in the prescription rate of HMV between Swedish counties, Laub et al. support the assertion that variations in individual enthusiasm may lead to geographical differences.^4^ Such a consequence based on the varying degrees of enthusiasm is hardly unique for HMV. Wisborg et al. found that “keeping the spirit high,” achieved through enthusiasm, was an important factor in order to explain why only a few Norwegian hospitals managed to implement and continue multidisciplinary trauma training.^11^ In our study, some respondents used the expression, “too much enthusiasm,” and thought this was the case in some high prevalence counties. This could lead to, among other things, prescription of HMV to patient groups without well-documented effects or who, for other reasons, should not receive the treatment. In two of the counties we interviewed, the prevalence was almost three times as high as the lowest prevalence county and the country average. Does this indicate that counties with very high prevalence have too many patients and that they prescribe HMV to patients without documented effect? We cannot answer this question since we did not study the indication criteria for treatment in all counties. Some respondents thought that national guidelines for HMV, which are currently being developed, will ensure that HMV will only be prescribed to patients with documented effect.

A highly specialized treatment offer, which to a large extent depends on a small number of enthusiastic individuals, was regarded as vulnerable. What happens when the enthusiast loses the enthusiasm or leaves? Maintaining the enthusiasm over a long period of time was described by respondents as challenging because the enthusiasts often meet resistance. A lack of acceptance and interest from colleagues and the administrative system are examples of such a resistance. If HMV was provided by a small number of enthusiasts, and was not accepted or officially established throughout the whole organization, there was an increased risk that the enthusiast would become burnt-out or that the offer of home mechanical ventilation would not be prioritized. Making difficult ethical decisions independently was also described as a heavy burden. In order to maintain the enthusiasm, the respondents emphasized the advantage of being in a multidisciplinary team. Multidisciplinary consensus was essential in making difficult ethical decisions.

Like Wisborg’s research,^11^ our research shows that enthusiasm alone is not sufficient for explaining the causes of geographical differences in practice. We found that high HMV competence among the multidisciplinary hospital team, particularly in complex and challenging treatment situations, was as significant as the competence level of the enthusiast. An example is establishing HMV to critically ill patients in need of tracheotomy, continuous ventilatory support and around-the-clock care. The enthusiasts were the driving force and role models in the development of this multidisciplinary competence. In our study, a large patient volume was described as a necessity in order to develop high competence. In Norway, there are more than 30 different hospital departments involved in establishing and following up HMV. Some of these have limited experience due to small patient volumes. In Denmark, with approximately 800,000 more inhabitants than Norway, there are two centers for establishing HMV.^12^ In order to develop high competence, and thereby ensure an equal geographical treatment offer, several respondents argued for centralizing the treatment in a few specialized centers in Norway. Fauroux et al. found that 86% of all children in France with long-term non-invasive home ventilation were followed up by four specialized pediatric centers.^13^ Due to the medical, technical and social complexity connected to the treatment of these patients, the authors recommended that the patients should be treated at a specialist center.

Respondents emphasized the importance of spreading competence about HMV within the different departments of the hospitals, in addition to the importance of health care services outside the hospital, to discover and recruit new patients. Spreading competence is not only essential for the recruitment of patients but also for the patients' experience within the health care system. Lindal et al. found that there is a lack of knowledge about HMV in the health care system, and that HMV patients feel they are met with uncertainty and fear among health care personnel.^14^ One of the hospitals we interviewed held courses in HMV for medical personnel from neighboring hospitals and the primary health care system. To spread competence, and thereby even out geographical differences, the Norwegian Medical Center for Home Mechanical Ventilation was established in 2002.^15^ This center has a network of colleagues who are employed at hospitals across the country. The respondents thought this measure was important, but that a lot of work still remains, especially in low prevalence counties.

Our study revealed that restrictive attitudes about HMV and the quality of life for patients among decision-making physicians, especially among neurologists, may influence whether or not the physician will offer HMV. These restrictive attitudes are similar to other studies.^6,16^ When considering the fact that one of these studies was published as early as 1992,^6^ it is obvious that such attitudes change slowly. Contrary to these restrictive attitudes, several studies show that HMV has a positive influence on the quality of life^17-19^ and that HMV patients live a full life with a large degree of autonomy and independence.^20^ The quality of life for patients with a tracheotomy is just as good as for those patients ventilated via a mask.^21^

## Research Limitations

We studied five of the approximately 30 total Norwegian hospital departments in which HMV is offered and established. The findings in qualitative research are not valid facts for the whole population.^22^ The aim of the grounded theory is to describe what is important to the participants in addition to clarifying and finding relevance and applicability to practice.^23^ However, there is reason to believe that our findings are representative of and relevant to HMV practice in other countries and all other Norwegian counties.

In our study, other factors that may lead to differences in HMV prevalence have not been studied systematically. In Sweden, a statistically significant connection between the relative number of pulmonologists and the number of home-ventilated patients was presented as a possible explanation to the huge differences between counties.^5^ The lack of exact data on the prevalence of HMV for all counties made such a calculation impossible in this study. Factors weighing heavily against such a connection are the two counties with the highest prevalence of HMV in our study (57 and 58/100,000) where the ratio between the number of HMV patients/100,000 and the number of pulmonologists/100,000 varied with a factor of two.

We have not studied whether variations in the organization of HMV between the counties and the health regions may explain the differences nor whether economic differences between the counties and the health regions may be of importance. However, the last point is unlikely because equal and fair distribution of economic resources is an important principle of the Norwegian public-financed health care system.

## Conclusion

Several factors seem to explain why the provision of HMV varies so widely within Norway. The most important factor seems to be the fact that enthusiasts with special competence and interest in HMV are unevenly distributed between hospitals. At a few hospitals in counties with high prevalence of HMV, such enthusiasts have established an environment focusing on these factors: high competence, spreading competence, and multidisciplinary collaboration. We describe this culture as wise enthusiasm. Individual attitudes about HMV, especially among neurologists, also seem to play a part in whether the patient is offered HMV. A lot of work remains in order to even out regional differences in the provision of HMV. Building competence in low-prevalence regions seems to be the most important measure. Centralizing into fewer competence centers should also be considered since many hospitals treat a very small number of patients. Data describing regional differences in the provision of HMV within countries is lacking. Further research is needed to identify these differences to ensure equality of provision of HMV.

## References

[1] Lloyd-Owen SJ Donaldson GC Ambrosino N Patterns of home mechanical ventilation use in Europe: results from the Eurovent survey. Eur Resp J 2005; 25: 1025-1031 10.1183/09031936.05.00066704 15929957

[2] Tollefsen E Gulsvik A Bakke P Fondenes O Prevalence of home ventilation therapy in Norway. Tidsskr Nor Laegeforen 9 A.D; 129: 2094-2097 10.4045/tidsskr.08.0716 19855446

[3] The Norwegian Council for Quality Improvement and Priority Setting in Health Care: Home Mechanical Ventilation. [document on the internet] [updated 2008 May 26]. [cited 2009 June 25]; Available from: http://www.kvalitetogprioritering.no/binary?id=2956 (accessed 25 June 2008).

[4] Laub M Berg S Midgren B Home mechanical ventilation in Sweden--inequalities within a homogenous health care system. Respir Med 2004; 98: 38-42 14959812 10.1016/j.rmed.2003.08.005

[5] Midgren B Schedin U Olofson J Artificial respiration at home seen in a 5-year perspective. Established treatment, but remarkable differences among the counties. Lakartidningen 2000; 97: 5483-5490 11192774

[6] Bach JR Ventilator use by muscular dystrophy association patients. Arch Phys Med Rehabil 1992; 73: 179-183 1543415

[7] Kitzinger J Qualitative research. Introducing focus groups. BMJ 1995; 311: 299-302 7633241 10.1136/bmj.311.7000.299 PMC2550365

[8] Powell RA Single HM Focus groups. Int J Qual Health Care 1996; 8: 499-504 9117204 10.1093/intqhc/8.5.499

[9] Morgan D Kreuger RA The focus group kit London: Sage, 1997

[10] Strauss A Corbin J Basics of qualitative research. Grounded Theory procedures and techniques, 2nd ed Thousand Oaks, CA: SAGE, 1998

[11] Wisborg T Brattebo G Keeping the spirit high: why trauma team training is (sometimes) implemented. Acta Anaesthesiol.Scand 2008; 52: 437-441 18205900 10.1111/j.1399-6576.2007.01539.x

[12] Midgren B Olofson J Harlid R Dellborg C Jacobsen E Norregaard O Home mechanical ventilation in Sweden, with reference to Danish experiences. Swedish Society of Chest Medicine. Respir Med 2000; 94: 135-138 10714418 10.1053/rmed.1999.0699

[13] Fauroux B Boffa C Desguerre I Estournet B Trang H Long-term noninvasive mechanical ventilation for children at home: a national survey. Pediatr Pulmonol 2003; 35: 119-125 12526073 10.1002/ppul.10237

[14] Lindahl B Sandman PO Rasmussen BH Meanings of living at home on a ventilator. Nurs Inq 2003; 10: 19-27 12622801 10.1046/j.1440-1800.2003.00160.x

[15] Norwegian Medical Center for Home Mechanical Ventilation: Information in English.[document on the internet] [updated 2003]. Available from: http://www.helse-bergen.no/avd/hjemmerespiratorbehandling/english/inenglish.htm (accessed 21 June 2008)

[16] Gibson B Long-term ventilation for patients with Duchenne muscular dystrophy: physicians' beliefs and practices. Chest 2001; 119: 940-946 11243978 10.1378/chest.119.3.940

[17] Bach JR Campagnolo DI Hoeman S Life satisfaction of individuals with Duchenne muscular dystrophy using long-term mechanical ventilatory support. Am J Phys Med Rehabil 1991; 70: 129-135 2039614 10.1097/00002060-199106000-00004

[18] Ballangrud R Bogsti WB Johansson IS Clients' experiences of living at home with a mechanical ventilator. J Adv Nurs 2009; 65: 425-434 19191941 10.1111/j.1365-2648.2008.04907.x

[19] Brooks D King A Tonack M Simson H Gould M Goldstein R User perspectives on issues that influence the quality of daily life of ventilator-assisted indi viduals with neuromuscular disorders. Can Respir J 2004; 11: 547-554 15611803 10.1155/2004/659147

[20] Dreyer PS Life on a ventilator Department of Nursing Science, University of Aarhus, Aarhus, 2004

[21] Markstrom A Sundell K Lysdahl M Andersson G Schedin U Klang B Quality-of-life evaluation of patients with neuromuscular and skeletal diseases treated with noninvasive and invasive home mechanical ventilation. Chest 2002; 122: 1695-1700 12426273 10.1378/chest.122.5.1695

[22] Malterud K Qualitative research: standards, challenges, and guidelines. Lancet 2001; 358: 483-488 11513933 10.1016/S0140-6736(01)05627-6

[23] Glacer B Theoretical sensitivity Mill Valley, CA: The Sociology Press, 1978

